# A Data Transformation Methodology to Create Findable, Accessible, Interoperable, and Reusable Health Data: Software Design, Development, and Evaluation Study

**DOI:** 10.2196/42822

**Published:** 2023-03-08

**Authors:** A Anil Sinaci, Mert Gencturk, Huseyin Alper Teoman, Gokce Banu Laleci Erturkmen, Celia Alvarez-Romero, Alicia Martinez-Garcia, Beatriz Poblador-Plou, Jonás Carmona-Pírez, Matthias Löbe, Carlos Luis Parra-Calderon

**Affiliations:** 1 Software Research & Development and Consultancy Corporation (SRDC) Cankaya Turkey; 2 Department of Computer Engineering Middle East Technical University Cankaya Turkey; 3 Group of Computational Health Informatics Institute of Biomedicine of Seville Virgen del Rocío University Hospital, Spanish National Research Council, University of Seville Seville Spain; 4 EpiChron Research Group Aragon Health Sciences Institute (IACS) Aragon Health Research Institute (IIS Aragon) Zaragoza Spain; 5 Institute for Medical Informatics, Statistics and Epidemiology (IMISE) University of Leipzig Leipzig Germany

**Keywords:** Health Level 7 Fast Healthcare Interoperability Resources, HL7 FHIR, Findable, Accessible, Interoperable, and Reusable principles, FAIR principles, health data sharing, health data transformation, secondary use

## Abstract

**Background:**

Sharing health data is challenging because of several technical, ethical, and regulatory issues. The Findable, Accessible, Interoperable, and Reusable (FAIR) guiding principles have been conceptualized to enable data interoperability. Many studies provide implementation guidelines, assessment metrics, and software to achieve FAIR-compliant data, especially for health data sets. Health Level 7 (HL7) Fast Healthcare Interoperability Resources (FHIR) is a health data content modeling and exchange standard.

**Objective:**

Our goal was to devise a new methodology to extract, transform, and load existing health data sets into HL7 FHIR repositories in line with FAIR principles, develop a Data Curation Tool to implement the methodology, and evaluate it on health data sets from 2 different but complementary institutions. We aimed to increase the level of compliance with FAIR principles of existing health data sets through standardization and facilitate health data sharing by eliminating the associated technical barriers.

**Methods:**

Our approach automatically processes the capabilities of a given FHIR end point and directs the user while configuring mappings according to the rules enforced by FHIR profile definitions. Code system mappings can be configured for terminology translations through automatic use of FHIR resources. The validity of the created FHIR resources can be automatically checked, and the software does not allow invalid resources to be persisted. At each stage of our data transformation methodology, we used particular FHIR-based techniques so that the resulting data set could be evaluated as FAIR. We performed a data-centric evaluation of our methodology on health data sets from 2 different institutions.

**Results:**

Through an intuitive graphical user interface, users are prompted to configure the mappings into FHIR resource types with respect to the restrictions of selected profiles. Once the mappings are developed, our approach can syntactically and semantically transform existing health data sets into HL7 FHIR without loss of data utility according to our privacy-concerned criteria. In addition to the mapped resource types, behind the scenes, we create additional FHIR resources to satisfy several FAIR criteria. According to the data maturity indicators and evaluation methods of the FAIR Data Maturity Model, we achieved the maximum level (level 5) for being Findable, Accessible, and Interoperable and level 3 for being Reusable.

**Conclusions:**

We developed and extensively evaluated our data transformation approach to unlock the value of existing health data residing in disparate data silos to make them available for sharing according to the FAIR principles. We showed that our method can successfully transform existing health data sets into HL7 FHIR without loss of data utility, and the result is FAIR in terms of the FAIR Data Maturity Model. We support institutional migration to HL7 FHIR, which not only leads to FAIR data sharing but also eases the integration with different research networks.

## Introduction

### Background

#### Health Data Sharing

The ability to share health data is bringing new horizons of research and innovation into view in many fields, such as integrated care, electronic health record (EHR) and personal health record management, and secondary use of EHRs for clinical research and public health studies. As digital health technology evolves, more and higher-quality data are accumulating within isolated databases of health care and health research institutions. Recent advancements in distributed data processing, big data analytics, and artificial intelligence have created a huge opportunity for analyzing the accumulated data addressing the increasing expectations of the population, which urges the need for health data sharing. However, sharing health data is challenging as a pile of technical, ethical, and regulatory issues are in place [1].

Technical challenges of health data sharing cover a broad spectrum of interoperability aspects such as data modeling, formatting, storing, accessing, and exchanging. Many efforts are being made to achieve interoperability through standardization, mostly for specific fields such as secondary use of health data for research or public health. A major approach is the adoption of Common Data Elements (CDEs) to standardize the content models and make them sufficiently common. The main obstacles hindering the interoperability in the CDE-based approach are the lack of governance of the CDEs, standardization of CDE interoperability, and associated implementations and software [2]. Another major and more practical approach is to build Common Data Models (CDMs). At present, there are 4 leading clinical research networks enforcing different CDMs for their participants: the Observational Health Data Sciences and Informatics (formerly Observational Medical Outcomes Partnership [OMOP]) [3,4], Informatics for Integrating Biology and the Bedside (i2b2) [5], PCORnet [6], and Sentinel [7]. Although interoperability is well achieved within each CDM network, it is laborious and troublesome for institutions to maintain their integration and infrastructure for each network [8]. That is, the interoperability challenge is pushed to another level by creating new isolated silos for clinical research using different content models and message exchange formats. There are efforts to harmonize these CDMs so that institutions can share health data even if they support different CDMs [8-11]. Furthermore, there are cases where available CDMs do not meet the interoperability requirements, and proprietary content model mappings and specific data integration architectures are developed [12], which further complicates the establishment of health data sharing architectures.

#### Findable, Accessible, Interoperable, and Reusable

The Findable, Accessible, Interoperable, and Reusable (FAIR) guiding principles have been conceptualized to enable machine-accessible and actionable data interoperability [13]. FAIR refers to a set of principles that should be perceived as guidelines; in other words, FAIR is not a standard and does not constrain implementation-related decisions. Worldwide, there has been a rapid uptake of the FAIR principles [14-17]. The biomedical research community is considerably leading FAIR adoption to share biomedical and health data sets of their research studies [18]. The application of the FAIR principles to biomedical data is of particular interest for researchers by developing domain-specific implementation flows, design decisions, and software [19,20]. With the growing interest in FAIR principles, tangible evaluation methods and metrics are being developed so that the level of compliance with FAIR principles (FAIRness) of implementations can be evaluated [21,22]. International alliances and research projects are providing implementation guidelines, workflows, assessment metrics, and software to achieve FAIR-compliant biomedical data [22,23]. Despite the substantial achievements in the FAIR-ification of biomedical data sets, research on the FAIR-ification of patient data residing in local EHRs and personal health records is still in its infancy. However, there is a workflow for sharing health care and health research data in line with the FAIR principles [24,25]. Already available standards and technologies in the EHR domain are being analyzed to show their adherence to the FAIR principles. The Health Level 7 (HL7) Fast Healthcare Interoperability Resources (FHIR) community initiated a project to develop an implementation guide on how the HL7 FHIR standard can be used to achieve FAIR health research data and how to fulfill the FAIR maturity indicators [26]. The openEHR framework was analyzed to show that its design principles handle the FAIR requirements [27]. As dominant clinical data modeling and exchange standards are seeking alignment with FAIR principles, we can expect that future versions of these standards will be more FAIR, and their implementers will inherently increase their level of FAIRness on health data.

#### HL7 FHIR Standard

HL7 has been developing and maintaining international standards for health data interoperability for decades. Owing to the wide adoption of HL7 version 2, HL7 version 3, and HL7 Clinical Document Architecture (CDA) [28], HL7 and the associated community have learned a lot from the feedback of the industry and academia. With the gained experience of previous standards and lessons learned, using the already available web service technologies, and relying on modern web development principles, the HL7 FHIR standard has been released as a health data content modeling and exchange standard [29]. Adopting a modular approach, health data entities such as patients, conditions, procedures, observations, and medications are modeled as FHIR resources. Although available FHIR resources primarily cover real-world health care entities and health data workflows, history has taught us that it is extremely challenging to come up with a single standard covering all possible requirements. Hence, FHIR uses the concept of profiles, with which existing FHIR resources can be adjusted to specific needs [30]. Cardinality constraints can be tightened, new fields can be added, existing ones can be restricted, or the use of value sets can be enforced among many other adjustment options that lead to the definition of profiles for the available FHIR resources. Starting from the building blocks of the FHIR standard to the definitions of the profiles, code systems, and value sets, all FHIR constructs can be processed by FHIR clients, and actions can be taken accordingly. This creates the opportunity for an FHIR client to start interacting with an FHIR server by reading its capabilities and acting accordingly by automatically processing the FHIR.

FHIR is well received by the health IT community. Starting with the Argonaut Project [31], several projects have been initiated to increase the adoption of FHIR by commercial EHR vendors [32]. There is strong private sector support and growing interest in FHIR by the big technology companies such as Apple, Google, IBM, and Microsoft, and by EHR vendors with the highest market share such as Epic and Cerner [33,34]. Support for HL7 FHIR is now required to meet the certification criteria of the Office of the National Coordinator for Health Information Technology [35]. European countries are placing the FHIR standard at the core of their eHealth applications. The National Health Service in the United Kingdom uses FHIR for the integration of their digital services [36], and Germany’s countrywide Medical Informatics Initiative uses FHIR to model their core data sets [37]. FHIR is welcomed by the open-source EHR community as well, such as OpenEMR and VistA, which were listed as the most promising open-source EHR systems in terms of the Office of the National Coordinator for Health Information Technology certification criteria [38]. Furthermore, FHIR also has influential support from academia; after its introduction, there has been a spike in the number of scientific articles referring to FHIR [39].

### Objectives

Acknowledging the importance of the FAIR principles in health data sharing and analyzing the growing interest in and adoption of HL7 FHIR, in this study, our goal was to develop a Data Curation Tool (DCT) around a new methodology to extract, transform, and load existing health care and health research data into HL7 FHIR repositories and evaluate the DCT on real-world health data from 2 different institutions. Our ultimate aim is to assist health data owners (health research–performing organizations) in creating FAIR data for the sake of health data sharing through a well-established and widely used standard in the health domain. Apart from the complete data transformation methodology, the principal novelty of our study lies in how we use the FHIR resources to meet each criterion of the FAIR principles.

Although health IT systems are starting to support the FHIR standard, it is a daunting task to transform the already existing data residing in legacy systems or simple spreadsheets into FHIR. Curating retrospective health data in conformance with FHIR requires extensive effort; it is time-consuming, and the domain experts carrying out this operation need deep technical knowledge of the FHIR standard. In line with the FAIR-ification workflow that we proposed in a previous study [25], we designed a practical methodology and developed a DCT so that the domain experts do not need to delve into the technical details of the FHIR standard and transform existing health data into FHIR through a computer desktop application with an intuitive graphical user interface (GUI). In addition to the configured resource types through mappings, our methodology automatically uses the additional FHIR resource types in a novel way so that all FAIR principles are met once the resource instances are written into the FHIR end point. We evaluated our methodology and the data transformation capability with real-world health care data from 2 different health institutions with respect to data utility and the level of FAIRness.

## Methods

### Overview

Adopting an iterative approach, we carried out the requirement elicitation and architecture design process for the DCT in close collaboration with researchers and experts from five different health care and health research organizations: (1) the Andalusian Health Service (SAS; Virgen del Rocío University Hospital) from Spain, (2) the Health Sciences Institute of Aragón (IACS) from Spain, (3) Geneva University Hospital from Switzerland, (4) the University of Porto from Portugal, and (5) Catholic University of the Sacred Heart from Italy. Researchers and developers from several other organizations (eg, from the authors’ affiliated organizations) also attended the collaboration activities during design and development. Following the security-privacy requirements and general design principles of the data curation and validation step of the FAIR-ification workflow [25], we started the development by providing mock-up GUIs to the researchers and hearing their comments in group interviews organized through web-based meeting sessions. Table 1 shows the total number of collaborators during these sessions categorized with respect to their FHIR knowledge. Most of them (10/22, 45%) were not aware of the FHIR standard, whereas some of them (6/22, 27%) had basic information about FHIR, such as it being a health care data standard for interoperability. We also worked with FHIR experts, mostly software engineers, during these sessions. In parallel, we analyzed these organizations’ existing data structure, storage format, and technology.

**Table 1:** Number of collaborators categorized according to their Fast Healthcare Interoperability Resources (FHIR) knowledge during the design and development (N=22).

	Categories of collaborators		
	No FHIR knowledge (n=10), n (%)	Basic knowledge of FHIR (n=6), n (%)	FHIR expert (with implementation experience; n=6), n (%)
SAS^a^	1 (10)	2 (33)	0 (0)
IACS^b^	1 (10)	1 (17)	0 (0)
UNIGE^c^	3 (30)	0 (0)	0 (0)
UP^d^	2 (20)	0 (0)	0 (0)
UCSC^e^	2 (20)	0 (0)	0 (0)
Others	1 (10)	3 (50)	6 (100)

^a^SAS: Andalusian Health Service.

^b^IACS: Health Sciences Institute of Aragón.

^c^UNIGE: Geneva University Hospital.

^d^UP: University of Porto.

^e^UCSC: Catholic University of the Sacred Heart.

**Figure 1:**
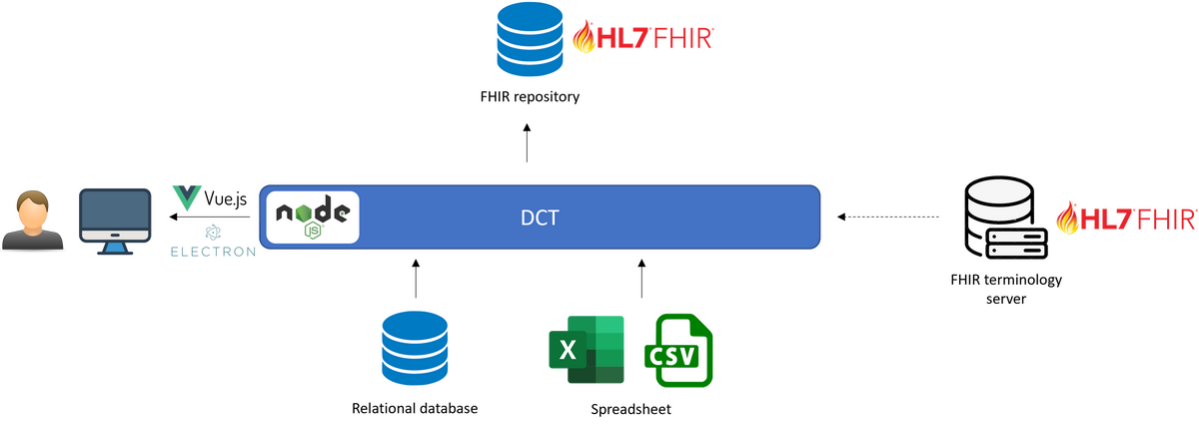
Placement of the Data Curation Tool (DCT) while transforming existing health data into Health Level 7 (HL7) Fast Healthcare Interoperability Resources (FHIR; brand icons were used for illustrative purposes).

As illustrated in Figure 1, we designed the DCT to be placed between health data sources and an FHIR repository and implemented it as a stand-alone desktop application. Considering the security-privacy requirements, we avoided providing a web-based solution so as to not migrate the sensitive health data to another server; the data transformation occurs on the computer that runs the DCT. However, we used web application development technologies such as Node.js (OpenJS Foundation), Vue.js, and Electron (OpenJS Foundation), which make the software easily convertible to a web application. The development of the software is open-source, and the source code together with the installation releases for major operating systems (Windows, macOS, and Linux) is available on GitHub [40].

At each step of our data transformation methodology, we applied specific techniques by using FHIR so that the resulting data set could be evaluated as FAIR, which constitutes the principal novelty of our work. In Textbox 1, we first summarize the steps of our methodology and then provide details of each step in different subsections.

**Summary of the steps of our methodology.**

- Access the Fast Healthcare Interoperability Resources (FHIR) end point and process the CapabilityStatement. If there are profile definitions, retrieve and parse them. Otherwise, proceed with the definitions of the standard resource types.
- Direct the user for the mappings. To address all principles of Findable, Accessible, Interoperable, and Reusable (FAIR), users are prompted to create valid FHIR resources with respect to profile definitions. For findability and accessibility, resource identifiers are generated through hashing, and each resource is directly accessible through URLs. Cross-references among the resources are handled through reference-specific mapping configurations.
- Guide the user for terminology translation. The FHIR application programming interface can be used to perform translations between terminology systems, either local, custom (eg, nationally standardized, international but customized, or completely proprietary), or international standards. This step specifically addresses the interoperability and reusability principles.
- Validate mappings by generating the FHIR resources and invoking the validation service of the FHIR end point. For the interoperability and reusability principles, our methodology checks each record from the source data before writing into the repository.
- Finish the transformation by writing the already generated FHIR resources. Behind the scenes, the Provenance and DocumentManifest resources are automatically created, and the generated FHIR resources are versioned so that the reusability principle is fully covered.

### FHIR Repository and Profiles

The DCT requires an FHIR server end point; hence, the user can proceed with mapping configuration and data transformation after the tool validates the FHIR end point. CapabilityStatement is an FHIR resource to describe the capabilities (ie, behaviors and features) of an FHIR server implementation. The DCT retrieves and processes the CapabilityStatement of the given FHIR repository and shows the user available FHIR resource types and supported profiles, including the supported operations for the resource types in the back end. The DCT is not bound by a specific FHIR implementation guide or set of profiles. Machine-processable profile definitions can be processed by the DCT, and then the rules exposed by the profile definitions are enforced on the user while they are configuring the mappings. As a result, invalid configurations are prohibited from the beginning.

During the evaluation of this study, we used onFHIR [41] as the FHIR repository, which is open-source, available on GitHub, and deployed within the firewalls of the organizations. Any valid FHIR profile definition from an implementation guide can be installed into onFHIR to enforce a set of rules. For this purpose, we configured the onFHIR installations using publicly available FHIR profiles [42]. With this setup, the DCT ensured valid FHIR resource creation with respect to the profile definitions supported by the connected FHIR repository.

### Mapping Configuration

**Figure 2:**
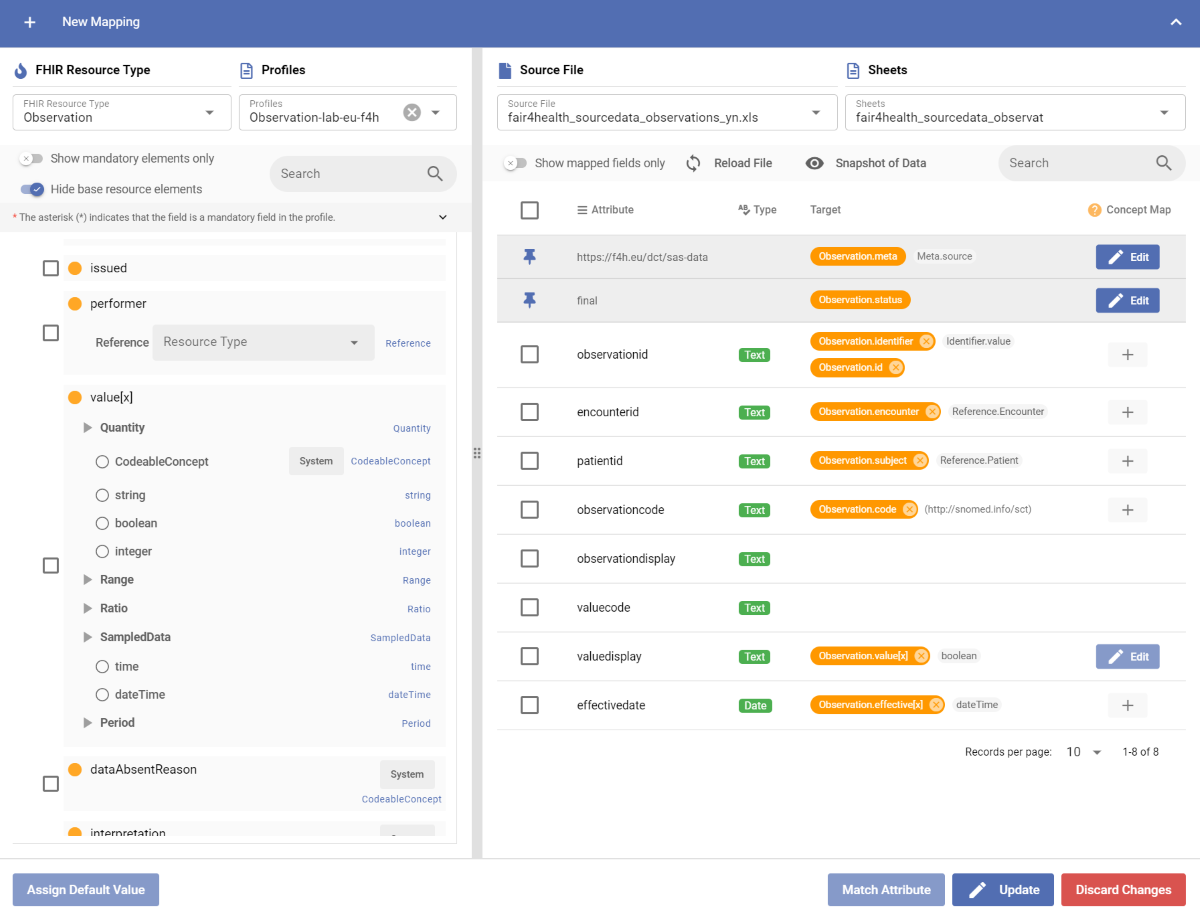
A screenshot showing a completed mapping to the Fast Healthcare Interoperability Resources (FHIR) Observation resource.

Once the CapabilityStatement of the FHIR repository is processed, the user can point to the CSV or Excel files or connect to a relational database to start configuring the mappings to the FHIR resource types from the source data structures with respect to the profile definitions. The user selects an FHIR resource type and a profile and then configures the mapping by matching the FHIR elements with the fields or columns of the selected sheet or table. Figure 2 shows a screenshot of the DCT in which a mapping to the FHIR Observation resource from a spreadsheet was performed by matching columns of the spreadsheet to the elements of the Observation resource. Unlike the traditional extract, transform, and load (ETL) software, our design puts the target of the mapping to the left and the source to the right. On the basis of the feedback that we received from the users of the tool, this design directs the user’s attention to the FHIR resource, and the user focuses on meeting the requirements of the selected FHIR resource and profile.

The user of the DCT is expected to have a basic understanding of health data entity types as categorized by HL7 FHIR so that they can select the correct resource types (eg, Observation, Condition, and MedicationStatement) for data tables or sheets to start the mapping process. In addition, the user needs to be aware of the general concept of a code system, value set, and coded values in their data set. Apart from that, no specific FHIR-related details such as the modeling details, restriction mechanisms, serialization formats, and application programming interface details need to be known by the user. The DCT provides the field names, short and long descriptions (when clicking on a field name), and data types; presents their cardinality and value set restrictions; and shows warnings in case a wrong mapping is configured into a resource field. Although these details are hidden, the user is expected to find the correct elements for each source field by analyzing the presented descriptions and restrictions.

**Figure 3:**
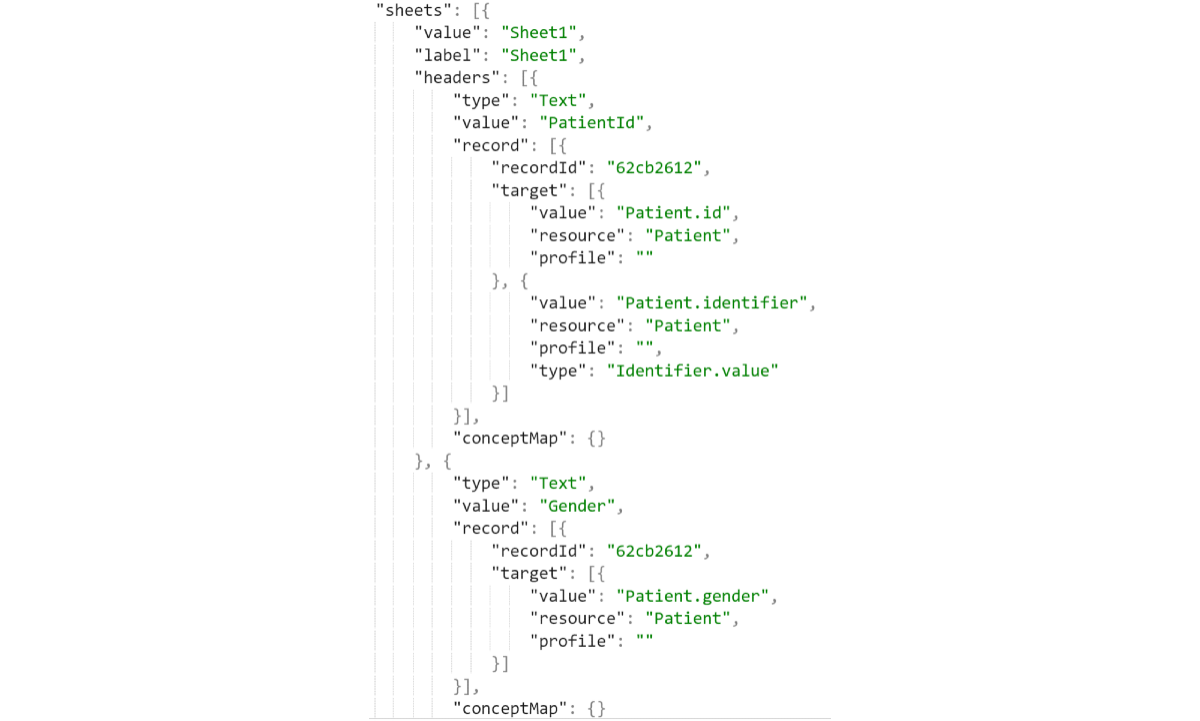
A snippet of the JSON-based mapping configuration that we generate underneath.

As the mappings are configured through the GUI, we generated a JSON-based mapping configuration that conforms to our internal mapping language. As shown in Figure 3, the GUI generates a mapping configuration, and then an engine executes these mappings during the validation and transformation. Instead of the FHIR Mapping Language [43], we designed an internal, proprietary mapping language as our methodology does not require many features of the FHIR Mapping Language, which increases development and configuration complexity. An important point to mention is that the referential integrity between FHIR resource instances is handled automatically if the source data have unique identification across records. As we use the same hashing algorithm to generate all *ID* fields of the resources and references between the resources, if a record holds a foreign key to another record and the mapping is created using the Reference selection (eg, *patientid* to *Observation.subject* through *Reference* in Figure 2), the integrity is reflected in the FHIR resources.

### Terminology Translation

Matching the FHIR elements and source data columns constitutes the syntactic transformation. In addition to that, our design handles the semantic transformation through code system mappings using an FHIR-based terminology server. At the start of the data curation process, the user can also point to a terminology server after pointing to the FHIR end point of the repository. Providing a terminology server is optional; data transformation may or may not need terminology translation based on the code systems used in the source data sets and the code systems enforced by the target FHIR profiles. The terminology translation mechanism of the DCT relies on FHIR again; CodeSystem, ValueSet, and ConceptMap resource types can be automatically processed by the DCT to handle code system mappings during the configuration and terminology translations during the data validation and transformation.

**Figure 4:**
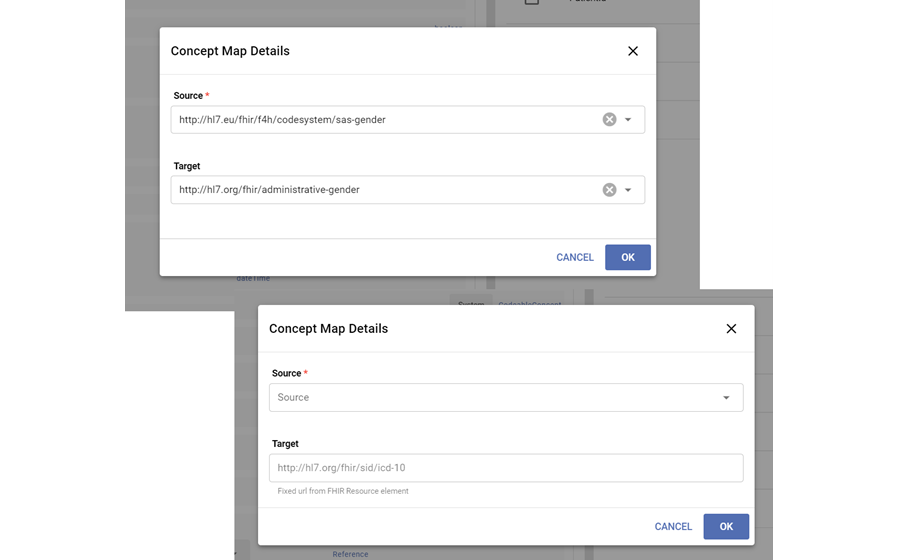
A screenshot showing 2 different code system mapping configurations for terminology translation.

After syntactically matching an FHIR element with a source data field, the user can configure the terminology translation for that matching if needed. For some data fields, source data can have proprietary or custom local code lists, whereas for some others, standard code systems can be in use. A good example of a custom code list is the gender field of the Patient resource as most of the data sets use their own codes to indicate the gender of a patient. If the target gender element under the Patient resource type enforces another code system, for example, the HL7 administrative gender codes, then our approach requires the mappings from the custom codes to the target code system to be put into and served from the connected FHIR terminology server. As shown in the upper part of Figure 4, our configuration can indicate the terminology translation from a local list of gender codes to the HL7 administrative gender codes while creating the associated Patient resources. In the lower part of Figure 4, another example is presented where the target code system is fixed as it is enforced by the FHIR profile definition, which means that the DCT must put International Classification of Diseases, 10th Revision (ICD-10), codes in that element while creating the Condition resources. If the source data set also uses ICD-10 as diagnosis codes, there is no need to configure a terminology translation. Otherwise, mappings can be configured from their local or custom codes, Systematized Nomenclature of Medicine–Clinical Terms (SNOMED-CT), or Medical Dictionary for Regulatory Activities (MedDRA) to ICD-10 or between different versions of the International Classification of Diseases if the connected FHIR terminology server provides the code mappings.

### Data Validation and Transformation

Creating valid FHIR resources depends not only on the correct mapping configurations but also on the source data themselves. While working on the mapping configurations, the user can see a snapshot of the data in that table or spreadsheet (eg, the first 10 rows) and test the validity of the created FHIR resources using those records. The DCT checks the validity of the resources with respect to the FHIR profile definitions that were selected while configuring those mappings. These include checking whether all mandatory elements are populated, cardinality restrictions are met, and value set enforcements together with the terminology translations are configured whenever necessary.

Once the mappings are completed, the user can run a full validation where the DCT creates all FHIR resources from the data and then validates them before saving them into the FHIR repository. In our methodology, the FHIR standard validation end point is used for this purpose. On the basis of the validation results, the user can go back and update the mappings as a single record in a spreadsheet or table may lead to an invalid FHIR resource. Otherwise, invalid resources will not be saved to the repository, whereas valid ones will. Although validating all records can be exhaustive and time-consuming, this ensures that the mapping configuration fits the nature of the source data and the already created resource instances can be directly used at the persistence step without additional processing. After the validation step, the transformed data can be written into the FHIR repository, and the curation process ends for the selected resources.

### Evaluation Setup

We evaluated our methodology and the DCT in terms of data transformation and FAIRness. We measured the data utility and assessed the level of FAIRness in the transformed data. We performed the evaluation on the health care data sets of 2 different health institutions in Spain: SAS and IACS. We chose these 2 institutions as they are complementary in the sense that they are from the same country with similar code systems for some fields and unstructured text fields in Spanish and yet they are different in the sense that SAS is a health care organization, whereas IACS is a health research institute with different source data structures (EHRs and research data registers, respectively) both syntactically and semantically.

Software deployment was performed separately for SAS and IACS according to the architecture depicted in Figure 1. At each institution, an expert who had a deep understanding of the data source’s content model carried out the data transformation using the DCT. Beforehand, document- and video-based user guides had been provided to the experts, and they had been trained on the DCT and the FAIR-ification process through web-based sessions. Although these sessions did not directly provide FHIR training, the basic concepts such as different resource types, profile definitions, code systems, and value sets were covered while discussing the features of the tool and methodology.

Textbox 2 summarizes which FHIR resource types were used and how they were used during the curation of the available health data entities in both SAS and IACS. During the evaluation, the Patient, Encounter, Observation, Condition, and MedicationStatement resource types were used to configure the mappings, meeting the requirements enforced by the profiles received from the FHIR repository. The Provenance and DocumentManifest resources were automatically created by the DCT behind the scenes.

**Resource types of the Fast Healthcare Interoperability Resources standard and their use descriptions during the evaluation.**

- Patient
  A Patient resource was created for each patient record in the source data to cover the basic demographics.
- Encounter
  An Encounter resource was created for each hospital admission and linked to the associated Patient resource.
- Observation
  An Observation resource was created for each vital sign observation, laboratory result, clinical assessment score, and social observation such as smoking history and status. The Observation was linked to the Patient resource and, if available, to the Encounter resource.
- Condition
  A Condition resource was created for each clinical condition and diagnosis and linked to the associated Patient and, if available, Encounter resources.
- MedicationStatement
  A MedicationStatement resource was created for each prescription and linked to the associated Patient and, if available, Encounter resources.
- Provenance
  A single Provenance resource was created for 1 data transformation session to represent the provenance information of the created resources during that session.
- DocumentManifest
  A single DocumentManifest resource was created for the data set to maintain the author and license information together with other metadata.

A methodological end-user evaluation of the DCT was performed in another study in which 16 experts were recruited from various organizations across Europe and assessed the usability of the tool through a series of questionnaires [43]. In the questionnaires, the experts evaluated the GUI functionalities of the tool against its requirements. Overall, the evaluation was performed successfully, with satisfactory results. In this study, in contrast, we evaluated our methodology in terms of data utility and FAIRness.

### Ethics Approval

Ethics approval for this study was obtained from the participating health research organizations (ie, Virgen del Rocío University Hospital as part of the SAS and IACS and Aragón Health Research Institute [1269-M1-20]) based on their regional regulations. Technical and organizational measures were defined to safeguard the rights and freedoms of the participants, including the data minimization principle, informed consent procedures, and information sheets. A data protection officer was appointed at each institution. To reinforce the appropriate coverage of these ethical aspects, at the beginning of the study, an external ethics advisory board was set up, which was responsible for reviewing deliverables, generating reports, and performing presentations to support the FAIR4Health Consortium.

## Results

### Data Utility

Data utility refers to the analytical completeness and validity of the transformed data. To measure data utility, we first compared the total number of entities on the source data with the same numbers on the target data, that is, on the FHIR resources, as shown in Table 2. In addition to the total numbers, to evaluate the correctness of the transformation in terms of detailed fields of curated entities, we developed 5 data utility criteria and calculated the numbers on both the source and transformed data sets. Table 3 presents the evaluation results for these privacy-concerned data utility criteria. We refer to them as *privacy-concerned* as, instead of providing the distribution of total numbers with respect to various categories (eg, number of male or female patients, distribution over each condition code, and laboratory result ranges), we agreed upon 5 criteria that we accepted as sufficient to evaluate the data utility while not violating the privacy rules of the data owner organizations according to the personal evaluations of their respective data protection officers. In this evaluation, we excluded the erroneous or missing data from the source tables so that we could accurately evaluate the data utility of our methodology. As our approach did not put the records that could not be validated into the FHIR repository, we informed the user of the details of those records that could not be transformed along with the reason (eg, a required field to generate the URL was missing or null or a numerical laboratory result value could not be parsed).

**Table 2:** Data utility results in terms of the total number of resources from the Fast Healthcare Interoperability Resources standard.

Data utility criterion	Value evaluated on source data, n		Value evaluated on transformed data, n	
	SAS^a^	IACS^b^	SAS	IACS
Patients	7873	7622	7873	7622
Admissions	11,954	12,106	11,954	12,106
Observations	73,817	11,999	73,817	11,999
Conditions	118,616	78,040	118,616	78,040
Prescriptions	42,164	70,880	42,164	70,880

^a^SAS: Andalusian Health Service.

^b^IACS: Health Sciences Institute of Aragón.

**Table 3:** Data utility results in terms of refined indicators covering all created resources from the Fast Healthcare Interoperability Resources standard.

Data utility criterion	Value evaluated on source data, n		Value evaluated on transformed data, n		Accuracy, %	
	SAS^a^	IACS^b^	SAS	IACS	SAS	IACS
Female patients with the most frequent 5 conditions in the data set	3067	2193	3067	2193	100	100
Patients diagnosed with COPD^c^ (ICD-10^d^ codes: J44, J44.0, J44.1, and J44.9)	1640	859	1640	859	100	100
Patients whose prescriptions included ≥5 medications	4089	4273	4089	4273	100	100
Readmissions within 30 days of discharge	919	1187	919	1187	100	100
Male patients whose hemoglobin (LOINC^e^ code: 718-7) values were between 138 and 172 g/L	381	—^f^	381	—	100	N/A^g^

^a^SAS: Andalusian Health Service.

^b^IACS: Health Sciences Institute of Aragón.

^c^COPD: chronic obstructive pulmonary disease.

^d^ICD-10: International Classification of Diseases, 10th Revision.

^e^LOINC: Logical Observation Identifiers Names and Codes.

^f^Not available.

^g^N/A: not applicable.

### FAIRness Level

**Figure 5:**
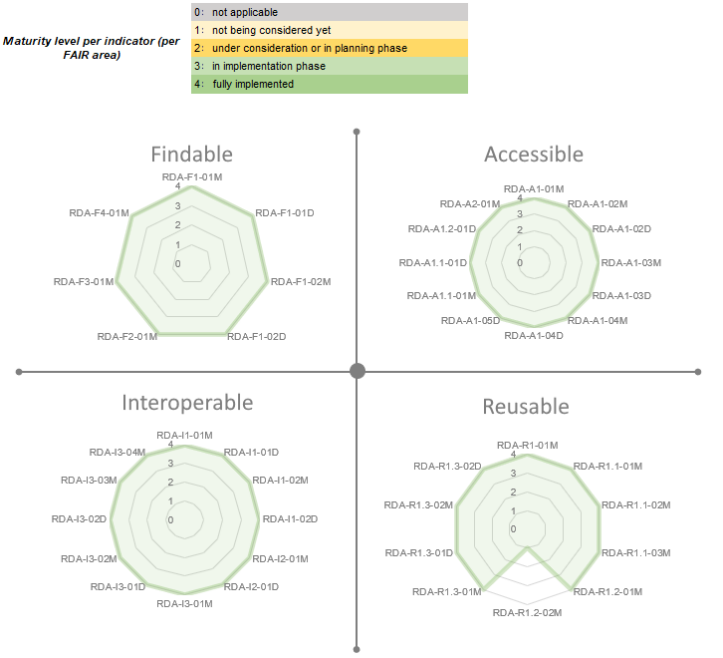
Progress of compliance with Findable, Accessible, Interoperable, and Reusable (FAIR) principles per FAIR maturity indicator.

To measure the level of FAIRness of the transformed data sets, we applied the maturity indicators and evaluation methods of the FAIR Data Maturity Model released by the Research Data Alliance (RDA) [44]. This model provides a set of indicators for each principle of FAIR, where each indicator has a unique identifier and is categorized as essential, important, or useful according to its priority. We evaluated the transformed data for each indicator and scored them as shown in Figure 5. While scoring each indicator, we considered the inherent capabilities of HL7 FHIR together with how we used the FHIR resources and profiles through the DCT. Although some indicators are directly covered by our methodology on FHIR use, some of them are indirectly handled through the standard and its implementation. Authentication of access (in the accessibility principle) is an example of an indicator that is indirectly covered by the FHIR standard and its implementation. In contrast, our approach ensures that data and metadata can be resolved and accessed and the metadata can remain available even after the data are no longer available (in the accessibility principle). In Multimedia Appendix 1, we provide a detailed analysis of how each indicator is covered by our methodology.

The maturity model makes a clear distinction between data and metadata while defining the indicators. Although it is not possible to draw a strict line between data and metadata in general, we designed the DCT to consider content modeling–related constructs of FHIR such as the profiles or the CapabilityStatement itself as metadata in addition to intrinsic metadata constructs such as the meta-element of the base FHIR resource (whose name is Resource) or resources such as Provenance and DocumentManifest, which are automatically created by the DCT during data transformation. Regarding the indicators of each FAIR principle, we summarize how we address them in Textbox 3.

**Definition of how we address each Findable, Accessible, Interoperable, and Reusable (FAIR) principle.**

- Findable
  The Data Curation Tool (DCT) assigns a globally unique, persistent, and resolvable identifier in the form of a URL to each resource to be created in the Fast Healthcare Interoperability Resources (FHIR) repository. The powerful search mechanism of FHIR and its onFHIR implementation provides a rich set of metadata to search through indexed fields.
- Accessible
  As the identifiers of the resources are URLs, they can be accessed manually or automatically through HTTP, which is a free protocol, and authentication and authorization can be enabled within HTTP if necessary.
- Interoperable
  The DCT creates references across FHIR resources using their URLs, such as referring from an Encounter resource to a Patient resource (referential integrity across FHIR resources). The knowledge representation of the content model is expressed using the FHIR standard through XML or JSON serializations. We used standard code systems such as Health Level 7 AdministrativeGender and International Classification of Diseases, 10th Revision (ICD-10), for associated data and metadata fields that address the FAIR-compliant vocabulary requirements of the respective indicators.
- Reusable
  The FHIR standard is a machine-understandable community standard, and each resource is associated with a Provenance resource as well as license information according to our transformation mechanism. Hence, all transformed data and their metadata comply with a community standard.

**Figure 6:**
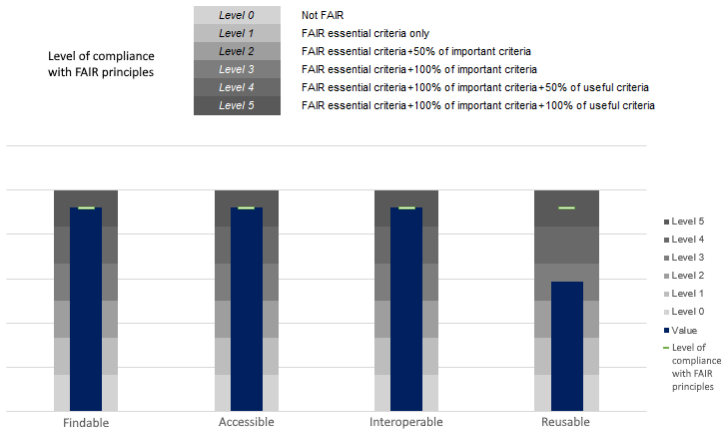
Level of compliance with Findable, Accessible, Interoperable, and Reusable (FAIR) principles per FAIR principle.

The evaluation results showed that only 1 indicator under the Reusable principle was not addressed by our implementation, as also shown in Figure 5. This unmet indicator is about providing provenance information with a cross-community language, and it is prioritized by the RDA as useful, not essential or important. Our approach uses a community-specific standard for health care, which is HL7 FHIR; hence, we evaluated that metric as “not being considered” for implementation. The resulting FAIRness level per the FAIR principle is presented in Figure 6, where all indicator criteria are fully met except for the one under the Reusable principle.

## Discussion

### Principal Findings

In this study, we demonstrated that our methodology and the DCT that we developed successfully enable the FAIR-ification of real-world health data—it is possible to translate the data into HL7 FHIR while meeting FAIR principles for health data sharing. The DCT enables domain experts to transform existing health data into FHIR via user-friendly GUIs. The evaluation results show that the utility and accuracy of the transformed data are preserved, whereas the maximum level of FAIRness (level 5) is achieved for the Findable, Accessible, and Interoperable principles and level 3 is achieved for the Reusable principle.

Following the wide adoption of the HL7 FHIR as a health data exchange standard, several efforts were initiated to transform data from legacy formats into FHIR. In the following paragraphs, we compare our approach with the major efforts and present the advantages of this study over them.

Clinical Asset Mapping Program for FHIR (CAMP FHIR) is an open-source software that transforms CDMs (such as i2b2, OMOP, and PCORnet) represented as relational database views to a set of predefined FHIR resources designed to represent clinical research data [8]. The mappings are defined as SQL scripts and run from the command line. The DCT not only supports relational databases but can also connect to the health data sources, which can be in various formats, including spreadsheets and CSV files. More importantly, the DCT provides GUIs for visually mapping source data to the FHIR, enabling subject matter experts to create the mapping definitions without dealing with scripting languages. Finally, instead of fixing the target model to a predefined set of FHIR resources designed for clinical research data, the DCT is flexible to process any FHIR profile represented via FHIR StructureDefinition; hence, it can be flexibly used in any other context (eg, protocol-driven research studies).

FHIR Converter [45] is an open-source project developed in .NET by Microsoft that can convert 2 types of source data, namely, HL7 version 2 and CDA, to FHIR resources via a predefined set of mappings. The DCT complements this tool by providing a user-friendly mapping tool for data sources that are not capable of exposing health data through the HL7 version 2 or CDA interfaces.

Firely FHIR Facade [46] is a paid plug-in for Firely FHIR Server that enables the execution of FHIR Mapping Language for transforming structured data (in HL7 version 2, Consolidated CDA, CSV, or custom FHIR resources) to and from FHIR resources. It does not provide a GUI to define the mappings between the FHIR logical models; hence, it is not suitable for use by nontechnical subject matter experts. It also does not support relational databases.

The limitations of our study can be summarized as follows. Health care data usually consist of missing and inaccurate parts, which makes it challenging to harmonize and use them in analytical processes. Our methodology allows for the use of FHIR profiles, which may impose several restrictions on the data fields. If such restrictive profiles are adopted and if a record does not comply with the restrictions of the profiles, then the record is logged for informative purposes and processing stops for that record; it is not persisted into the FHIR repository. On the basis of the nature of the source data set and the required level of harmonization at the end of the curation, the profiles can be adjusted to handle the level of inaccuracy and incompleteness of the records.

We collaborated with 5 different health care or health research organizations during the design and development of the methodology and the DCT. However, we performed the evaluation with 2 of them, which can be considered a limitation of this study. Although these 2 institutions provided large data sets for the evaluation, our study is still limited considering the vast number of entities being used in digital health systems. Moreover, information provided by patients using telemedicine devices or other sources was not included.

We showed that our approach met the data transformation requirements of 2 different health organizations, whereas we worked with 5 different organizations from 4 different countries during the design and development. Although major requirements from different health organizations can be met with our methodology, we believe that our work can still be improved with complex mapping and transformation requirements from different health research–performing organizations. The DCT may need to combine source fields through some simple or complex functions while mapping to a single target field. Alternatively, one source record can lead to multiple FHIR resource instances. Furthermore, our study was limited by the relatively small data sets of the participating organizations with respect to data processing throughput. Although it successfully validates our methodology, the DCT can be overwhelmed by very large data sets as it is a stand-alone desktop application. However, our data transformation methodology can be implemented using distributed data processing techniques to cope with large volumes of health data sets. As future work, we aim to extend the implementation to handle very large or distributed data sets while working on an improved version of our internal mapping language to process complex joint operations among different data sources. Our methodology also needs to be validated with more institutions from different countries to cover the many other requirements of different health care and health research settings. In the future, we will also work on the redesign and more formal definition and implementation of the internal mapping language to make it more external and publishable.

The DCT and our use of FHIR support the institutional migration to HL7 FHIR, which not only increases the level of FAIRness but also eases the adoption of *SMART on FHIR* applications [47]. This enables health care institutions to readily use a wide range of third-party applications such as decision support services, risk calculators, and charts [48]. Our method and DCT also contribute to the integration and harmonization with clinical research models for the secondary use of health care data. The Biomedical Research Integrated Domain Group (BRIDG) Model [49,50] is an important effort for enabling secondary use of health care data that was collaboratively developed over the past 15 years by the Food and Drug Administration, National Cancer Institute, Clinical Data Interchange Standards Consortium, and HL7 to enable protocol-driven research by providing a shared view of the semantics for basic, preclinical, clinical, and translational research and its associated regulatory artifacts. Our approach will lead to easy adoption of the BRIDG Model; it will be possible to set the BRIDG FHIR resources [51] as the target FHIR model to map the existing EHR data to the BRIDG Model. The DCT supports the implementation of the recent vision statement from the HL7 Vulcan Accelerator Project [52], which aims to “fully integrate research into the delivery of healthcare by streamlining data collection and exchange into a singular process*”* via HL7 FHIR as the data exchange mechanism. The Observational Health Data Sciences and Informatics and HL7 announced a collaboration to enable the sharing of information in clinical care and observational research [53,54] by supporting mappings between OMOP and HL7 FHIR. The DCT can be positioned as an enabler of this effort, facilitating mappings between the OMOP model and custom-developed HL7 FHIR profiles for clinical research studies.

### Conclusions

In this paper, we presented a DCT and a data transformation approach to unlock the value of existing health data residing in disparate data silos to make them available for sharing as FHIR resources in line with the FAIR principles. Starting from the requirement analysis, we adopted an agile approach while working on the GUI mock-ups, software design, and implementation to iteratively return to the end users and develop the DCT accordingly. We performed an extensive evaluation using real-world data from 2 different but complementary Spanish institutions, with SAS being a hospital and IACS being a health research institute. We evaluated the utility and accuracy of the transformed data using several criteria that were created respecting the privacy concerns of the data owners. We showed that the DCT can successfully transform existing health data sets into HL7 FHIR without loss of data utility. In addition, we performed a methodological evaluation of the level of FAIRness of the transformed data using the data maturity indicators and evaluation methods of the FAIR Data Maturity Model released by the RDA. We concluded that our results achieved the maximum level of FAIRness (level 5) for the Findable, Accessible, and Interoperable principles, whereas we achieved level 3 for the Reusable principle, which covers the essential and important criteria of the FAIR metrics. The evaluation results showed that the DCT can make health data FAIR by transforming them into HL7 FHIR.

## Appendix

Multimedia Appendix 1 The Findable, Accessible, Interoperable, and Reusable Data Maturity Model as published by the Research Data Alliance and our evaluation score for each indicator are shown in Table S1. For each principle (Findable, Accessible, Interoperable, and Reusable), there is a unique indicator identifier, a description of the indicator, its priority, and the score of the evaluation in the metric column.

## Abbreviations

BRIDG: Biomedical Research Integrated Domain Group
CAMP FHIR: Clinical Asset Mapping Program for Fast Healthcare Interoperability Resources
CDA: Clinical Document Architecture
CDE: Common Data Element
CDM: Common Data Model
DCT: Data Curation Tool
EHR: electronic health record
ETL: extract, transform, and load
FAIR: Findable, Accessible, Interoperable, and Reusable
FAIRness: compliance with Findable, Accessible, Interoperable, and Reusable principles
FHIR: Fast Healthcare Interoperability Resources
GUI: graphical user interface
HL7: Health Level 7
i2b2: Informatics for Integrating Biology and the Bedside
IACS: Health Sciences Institute of Aragón
ICD-10: International Classification of Diseases, 10th Revision
MedDRA: Medical Dictionary for Regulatory Activities
OMOP: Observational Medical Outcomes Partnership
RDA: Research Data Alliance
SAS: Andalusian Health Service
SNOMED-CT: Systematized Nomenclature of Medicine–Clinical Terms

## Data Availability

The data sets generated and analyzed during this study are not publicly available as they contain information that could compromise research participant privacy and consent.
