# Supplementary material for: A Data Transformation Methodology to Create Findable, Accessible, Interoperable, and Reusable Health Data: Software Design, Development, and Evaluation Study
Source: J Med Internet Res. 2023 Mar 8;25:e42822. doi: 10.2196/42822 (PMC10034606; doi:10.2196/42822)
Supplement: Multimedia Appendix 1 [file jmir_v25i1e42822_app1.docx]

# APPENDIX

FAIRness Data Maturity Model as published by RDA and our evaluation score for each indicator is presented in Table A. For each principle (Findable, Accessible, Interoperable, Reusable), there is a unique indicator identifier, a description of the indicator, its priority, and the score for evaluation in the metric column.

Table A – FAIRness Data Maturity Model and our evaluation score for each indicator.

| PRINCIPLE | INDICATOR_ID | INDICATOR | PRIORITY | METRIC* |
| --- | --- | --- | --- | --- |
| F1 | RDA-F1-01M | Metadata is identified by a persistent identifier | Essential | 4 |
| F1 | RDA-F1-01D | Data is identified by a persistent identifier | Essential | 4 |
| F1 | RDA-F1-02M | Metadata is identified by a globally unique identifier | Essential | 4 |
| F1 | RDA-F1-02D | Data is identified by a globally unique identifier | Essential | 4 |
| F2 | RDA-F2-01M | Rich metadata is provided to allow discovery | Essential | 4 |
| F3 | RDA-F3-01M | Metadata includes the identifier for the data | Essential | 4 |
| F4 | RDA-F4-01M | Metadata is offered in such a way that it can be harvested and indexed | Essential | 4 |
| A1 | RDA-A1-01M | Metadata contains information to enable the user to get access to the data | Important | 4 |
| A1 | RDA-A1-02M | Metadata can be accessed manually (i.e. with human intervention) | Essential | 4 |
| A1 | RDA-A1-02D | Data can be accessed manually (i.e. with human intervention) | Essential | 4 |
| A1 | RDA-A1-03M | Metadata identifier resolves to a metadata record | Essential | 4 |
| A1 | RDA-A1-03D | Data identifier resolves to a digital object | Essential | 4 |
| A1 | RDA-A1-04M | Metadata is accessed through standardised protocol | Essential | 4 |
| A1 | RDA-A1-04D | Data is accessible through standardised protocol | Essential | 4 |
| A1 | RDA-A1-05D | Data can be accessed automatically (i.e. by a computer program) | Important | 4 |
| A1.1 | RDA-A1.1-01M | Metadata is accessible through a free access protocol | Essential | 4 |
| A1.1 | RDA-A1.1-01D | Data is accessible through a free access protocol | Important | 4 |
| A1.2 | RDA-A1.2-01D | Data is accessible through an access protocol that supports authentication and authorisation | Useful | 4 |
| A2 | RDA-A2-01M | Metadata is guaranteed to remain available after data is no longer available | Essential | 4 |
| I1 | RDA-I1-01M | Metadata uses knowledge representation expressed in standardised format | Important | 4 |
| I1 | RDA-I1-01D | Data uses knowledge representation expressed in standardised format | Important | 4 |
| I1 | RDA-I1-02M | Metadata uses machine-understandable knowledge representation | Important | 4 |
| I1 | RDA-I1-02D | Data uses machine-understandable knowledge representation | Important | 4 |
| I2 | RDA-I2-01M | Metadata uses FAIR-compliant vocabularies | Important | 4 |
| I2 | RDA-I2-01D | Data uses FAIR-compliant vocabularies | Useful | 4 |
| I3 | RDA-I3-01M | Metadata includes references to other metadata | Important | 4 |
| I3 | RDA-I3-01D | Data includes references to other data | Useful | 4 |
| I3 | RDA-I3-02M | Metadata includes references to other data | Useful | 4 |
| I3 | RDA-I3-02D | Data includes qualified references to other data | Useful | 4 |
| I3 | RDA-I3-03M | Metadata includes qualified references to other metadata | Important | 4 |
| I3 | RDA-I3-04M | Metadata include qualified references to other data | Useful | 4 |
| R1 | RDA-R1-01M | Plurality of accurate and relevant attributes are provided to allow reuse | Essential | 4 |
| R1.1 | RDA-R1.1-01M | Metadata includes information about the licence under which the data can be reused | Essential | 4 |
| R1.1 | RDA-R1.1-02M | Metadata refers to a standard reuse licence | Important | 4 |
| R1.1 | RDA-R1.1-03M | Metadata refers to a machine-understandable reuse licence | Important | 4 |
| R1.2 | RDA-R1.2-01M | Metadata includes provenance information according to community-specific standards | Important | 4 |
| R1.2 | RDA-R1.2-02M | Metadata includes provenance information according to a cross-community language | Useful | 1 |
| R1.3 | RDA-R1.3-01M | Metadata complies with a community standard | Essential | 4 |
| R1.3 | RDA-R1.3-01D | Data complies with a community standard | Essential | 4 |
| R1.3 | RDA-R1.3-02M | Metadata is expressed in compliance with a machine-understandable community standard | Essential | 4 |
| R1.3 | RDA-R1.3-02D | Data is expressed in compliance with a machine-understandable community standard | Important | 4 |

* 1 – not being considered yet, 2 – under consideration or in planning phase, 3 – in implementation phase, 4 – fully implemented

In Table B, we give explanations from our methodology about how we meet the challenges of the indicators.

Table B – Brief explanation of our solution to meet the challenges of each indicator.

| **INDICATOR_ID** | **OUR SOLUTION** |
| --- | --- |
| RDA-F1-01M | Alongside the resources such as Patient, Condition and Observation (data), definition resources such as StructureDefinition, CodeSystem and ValueSet (metadata) are first class resources in FHIR. Hence when they are created, we generate hashed resource identifiers (using the mappings configured by the users together with other column values of each record) concatenated with the deployment host of the FHIR endpoint which makes the identifiers persistent and globally unique. |
| RDA-F1-01D |  |
| RDA-F1-02M |  |
| RDA-F1-02D |  |
| RDA-F2-01M | We define various search parameters for each resource type to make them discoverable through FHIR search. This allows fine-grained search statements (e.g., female patients above an age and have a certain condition) for inclusion/exclusion criteria during search. On dataset level (for collection of resources), we create associated resources such as Provenance and DocumentManifest and add many descriptive statistics such as total number of records, average, min, max for appropriate fields. |
| RDA-F3-01M | Metadata within the resources are discoverable through FHIR search mechanism and directly within the data resources. Individual resources that we create as supportive metadata contains the discoverable identifiers through FHIR Reference elements. |
| RDA-F4-01M | We keep the metadata within the FHIR resources attached to data in addition to separate metadata resources which are again FHIR resource instances, and they can all be indexed through indexing configuration of the utilized FHIR implementation. |
| RDA-A1-01M | We utilize FHIR Reference elements to establish cross-references between FHIR resources, hence metadata contains discoverable references to data items. |
| RDA-A1-02M | All FHIR resources (data and metadata) can be accessed through FHIR REST API and manual access is possible through REST clients (e.g., internet browsers) used by humans. |
| RDA-A1-02D |  |
| RDA-A1-03M | Metadata and data records are FHIR resources or elements under FHIR resources and FHIR identifiers (URLs) resolve to their resource instances. |
| RDA-A1-03D |  |
| RDA-A1-04M | HL7 FHIR provides an Application Programming Interface (API) over HTTP using REST principles and the payload of the HTTP communication can be XML or JSON serialization of the FHIR resources. Hence, data and metadata is accessible through a free, standardized protocol and the API can be utilized by client computer programs. HTTP communication can be secured through well-established authentication and authorization mechanisms which are referred and suggested by HL7 FHIR to be implemented. onFHIR repository that we utilized in our study provides this security machinery. |
| RDA-A1-04D |  |
| RDA-A1-05D |  |
| RDA-A1.1-01M |  |
| RDA-A1.1-01D |  |
| RDA-A1.2-01D |  |
| RDA-A2-01M | We keep dataset-level metadata as separate FHIR resource instances having cross-references to the data resources. Hence, metadata is guaranteed to remain available after data resources are no longer available. |
| RDA-I1-01M | Being a standard for health data, HL7 FHIR inherently provides a machine-understandable knowledge representation expressed in a standard format. We further utilize FHIR profiling to add more knowledge such as restricting the use of value sets and performing validation accordingly. |
| RDA-I1-01D |  |
| RDA-I1-02M |  |
| RDA-I1-02D |  |
| RDA-I2-01M | Aligned with the recommendations of HL7 FHIR, we utilize existing healthcare vocabularies by enforcing their use in FHIR profile definitions. Apart from HL7’s code systems and value sets; ICD-10, SNOMED and ATC are major vocabularies that we utilize. |
| RDA-I2-01D |  |
| RDA-I3-01M | We establish the cross-references among the FHIR resources created using FHIR Reference elements. For example, each Condition resource refers to a Patient resource or each Provenance resource refers to a list of resources which are related with that Provenance information. Hence, if configured correctly during the mappings, all relations are established while generating the FHIR resources.  Metadata includes references to other metadata |
| RDA-I3-01D |  |
| RDA-I3-02M |  |
| RDA-I3-02D |  |
| RDA-I3-03M |  |
| RDA-I3-04M |  |
| RDA-R1-01M | Apart from the enforced cross-references and use of controlled vocabularies through FHIR profiling, we add descriptive statistics to the DocumentManifest resources to provide detailed information about the resources (e.g, total number of records; average, min, max values for numerical fields etc.) within that manifest, particularly giving insights about the eligibility criteria. |
| RDA-R1.1-01M | At the end of the data curation process, the DCT enforces the user to provide a License for that dataset being curated. We offer a list of Creative Commons licenses so that the user can directly select one of the appropriate, but we allow the user to write her own proprietary license also. The License information is put under the DocumentManifest resource and it is referenced through its URL which can be accessed and processed by machines. |
| RDA-R1.1-02M |  |
| RDA-R1.1-03M |  |
| RDA-R1.2-01M | We utilize FHIR Provenance resource to record the provenance information as separate FHIR resources. |
| RDA-R1.2-02M | Although the authors do not have unanimity, we do not categorize HL7 FHIR as a cross-community language. That’s why we scored this indicator as “1 – not being considered yet”. |
| RDA-R1.3-01M | We categorize HL7 FHIR as a community standard, hence data and metadata comply with a machine-understandable community standard. |
| RDA-R1.3-01D |  |
| RDA-R1.3-02M |  |
| RDA-R1.3-02D |  |
